# Buprenorphine Injection Among Rural Persons Who Inject Drugs

**DOI:** 10.1001/jamanetworkopen.2024.50108

**Published:** 2024-12-11

**Authors:** Kaitlin A. Zinsli, Elenore P. Bhatraju, Judith Feinberg, Thomas J. Stopka, Peter D. Friedmann, William Zule, Hannah L. F. Cooper, April M. Young, P. Todd Korthuis, Mai T. Pho, Wiley D. Jenkins, William C. Miller, Vivian F. Go, David W. Seal, Ryan P. Westergaard, Rob J. Fredericksen, Joseph A. Delaney, Judith I. Tsui

**Affiliations:** 1 Department of Epidemiology, University of Washington, Seattle; 2 Department of Medicine, University of Washington School of Medicine, Seattle; 3 West Virginia University School of Medicine, Morgantown; 4 Department of Public Health and Community Medicine, Tufts University School of Medicine, Boston, Massachusetts; 5 Department of Medicine, University of Massachusetts Medical School-Baystate, Springfield; 6 Substance Abuse Treatment Evaluations and Interventions Program, RTI International, Research Triangle Park, North Carolina; 7 Rollins School of Public Health, Emory University, Atlanta, Georgia; 8 Department of Epidemiology and Environmental Health, University of Kentucky, Lexington; 9 Department of Medicine, Oregon Health Sciences University, Portland; 10 Department of Medicine, University of Chicago Medical Center, Chicago, Illinois; 11 Department of Public Health Sciences, Clemson University, Clemson, South Carolina; 12 Department of Epidemiology, Gillings School of Global Public Health, University of North Carolina, Chapel Hill; 13 Department of Health Behavior, Gillings School of Global Public Health, University of North Carolina, Chapel Hill; 14 Department of Social, Behavioral, and Population Sciences, Tulane University School of Public Health and Tropical Medicine, New Orleans, Louisiana; 15 University of Wisconsin School of Medicine and Public Health, Madison

## Abstract

This cross-sectional study investigates buprenorphine injection, including factors associated with this use, among rural individuals who inject drugs.

## Introduction

Prescribed buprenorphine is a mainstay of opioid use disorder (OUD) treatment. Illicit buprenorphine injection has not been robustly described in the US.^1^ In 1 study of US patients seeking OUD treatment, 26% reported injecting buprenorphine among 58% reporting nonprescription buprenorphine use.^2^ OUD treatment barriers that may contribute to nonprescribed buprenorphine use^3^ are more pronounced in rural areas, where access to medications for OUD is less robust than in urban areas.^4^ We aimed to describe buprenorphine injection by rural people who inject drugs to inform patient-clinician discussions and public health interventions.

## Methods

This exploratory, cross-sectional, secondary analysis used data from the Rural Opioid Initiative, a multisite, federally funded project in 65 rural counties in 10 US states. All study sites obtained local institutional review board approval and informed consent, which extend to this study. People who use drugs were surveyed from January 2018 to March 2020 regarding substance use and associated health behaviors.^5^ Eligibility included past 30-day opioid use or any illicit drug injection. We excluded participants not reporting past 30-day injection drug use. Survey measures included questions on substance use, socioeconomic status, drug injection practices, substance use treatment, health care use, engagement in harm reduction services, and sexually transmitted or blood-borne infection diagnoses. The primary outcome was reported injection of sublingual buprenorphine in the past 30 days. We calculated unadjusted odds ratios (ORs) and 95% CIs to assess associations between survey measures and our outcome. This report follows the STROBE reporting guideline. We used Stata version 18 (StataCorp) and completed analyses August 2023 to May 2024.

## Results

Among 2369 study participants who injected drugs (mean [SD] age, 35.7 [10.0] years, 1003 cisgender women [42.4%]), 642 individuals (27.1%) reported injecting buprenorphine in the past 30 days. Women had lower odds of this outcome compared with men (OR, 0.83; 95% CI, 0.69-0.99), as did participants who had completed some college or trade school compared with those who completed less education (OR, 0.72; 95% CI, 0.58-0.88). Participants reporting being in jail or prison at least 1 day in the past month compared with those who had not had higher odds of reporting past 30-day buprenorphine injection (OR, 1.24; 95% CI, 1.03-1.49) (Table 1). Buprenorphine injection was more common among individuals reporting receiving buprenorphine from “a doctor or program” in the past 30 days (OR, 2.42; 95% CI, 1.87-3.13) or being unable to access buprenorphine from a health care professional in the past 6 months (OR, 1.65; 95% CI, 1.32-2.06). Participants who injected buprenorphine more commonly reported injecting drugs daily vs monthly (OR, 2.76; 95% CI,1.99-3.83), sharing syringes (OR, 1.65; 95% CI, 1.37-1.99), sharing injection equipment (OR, 1.92; 95% CI, 1.59-2.32), having condomless sex (OR, 1.32; 95% CI, 1.07-1.63), and consuming 4 to 5 or more alcoholic drinks (OR, 1.69; 95% CI, 1.40-2.03) in the past 30 days. People who injected buprenorphine more commonly preferred synthetic opioids (U-47700, U4, or pink) as their drug of choice (OR, 6.88; 95% CI, 2.45-19.27), had ever experienced an overdose (OR, 1.58; 95% CI, 1.31-1.91), and had a history of hepatitis C (OR, 1.24; 95% CI, 1.02-1.50) (Table 1; Table 2).

**Table 1.** Demographic Characteristics

Characteristic	Participants, No. (%) (N = ‭2369)^a^		Unadjusted OR (95% CI)
	Injected buprenorphine past 30 d (n = 642)	Did not inject buprenorphine past 30 d (n = 1727)	
Age (mean, SD), y	34.7 (9.4)	36.1 (10.2)	0.99 (0.98-1.00)^b^
Gender^c^			
Cisgender man	389 (60.6)	963 (55.8)	1 [Reference]
Cisgender woman	251 (39.1)	752 (43.6)	0.83 (0.69-0.99)
Transgender or other	2 (0.3)	11 (0.6)	0.45 (0.10-2.04)
Race and ethnicity^d^			
American Indian	43 (6.7)	128 (7.4)	0.88 (0.62-1.27)
Black or African American	12 (1.9)	51 (3.0)	0.62 (0.33-1.17)
Hispanic	18 (2.8)	62 (3.6)	0.76 (0.45-1.30)
White	545 (84.9)	1433 (82.9)	1 [Reference]
Other or unknown	24 (3.7)	53 (3.1)	1.19 (0.73-1.95)
Education			
≤High school	472 (73.6)	1160 (67.3)	1 [Reference]
Some college or trade school	154 (24.0)	528 (30.6)	0.72 (0.58-0.88)
≥College graduate	15 (2.3)	37 (2.1)	1.00 (0.54-1.83)
Income			
Full- or part-time work	173 (28.0)	452 (28.1)	1 [Reference]
Supported by someone	100 (16.2)	268 (16.6)	0.97 (0.73-1.30)
Retirement, public assistance, or disability check	85 (13.8)	292 (18.1)	0.76 (0.56-1.03)
Selling sex or drugs or stealing	90 (14.5)	187 (11.6)	1.26 (0.93-1.71)
Multiple	170 (27.5)	412 (25.6)	1.08 (0.84-1.39)
Experienced homelessness past 6 mo	349 (55.0)	953 (56.1)	0.96 (0.80-1.15)
In jail or prison ≥1 day past 6 mo	297 (47.1)	697 (41.7)	1.24 (1.03-1.49)
Current health insurance	463 (75.0)	1271 (75.8)	0.96 (0.77-1.19)
HCV diagnosis ever	244 (39.8)	578 (34.8)	1.24 (1.02-1.50)
HIV diagnosis ever	9 (1.4)	20 (1.2)	1.22 (0.55-2.70)

Abbreviations: HCV, hepatitis C virus; OR, odds ratio.

^a^ Participants were recruited across 10 US states, including Illinois, Kentucky, New England (New Hampshire, Vermont, and Massachusetts), North Carolina, Oregon, Ohio, West Virginia, and Wisconsin.

^b^ The OR for age was per 1-year increase.

^c^ The transgender or other category includes anyone that selected the *Other* option for the question asking about participant gender on the survey instrument.

^d^ Race and ethnicity were captured via self-report by participants using the study survey instrument. Race and Hispanic ethnicity are mutually exclusive. The other or unknown category includes individuals with unknown race and ethnicity or who did not select Hispanic ethnicity but selected 1 of the following other survey options: African; Alaska Native; Asian, Pacific Islander, or Native Hawaiian; mixed race; or other. Race and ethnicity were assessed to highlight differences that reflect health inequities or disparities in health from unjust structural practices and policies, such as those due to systemic racism.

**Table 2.** Behavioral Characteristics

Characteristic	Participants, No. (%) (N = ‭2369)^a^		Unadjusted OR (95% CI)
	Injected buprenorphine past 30 d (n = 642)	Did not inject buprenorphine past 30 d (n = 1727)	
Frequency of injecting drugs past 30 d			
At least monthly (<weekly)	47 (7.3)	278 (16.3)	1 [Reference]
At least weekly (<daily)	89 (13.9)	349 (20.4)	1.51 (1.02-2.22)
Daily or more	504 (78.8)	1080 (63.3)	2.76 (1.99-3.83)
Syringe sharing past 30 d	310 (50.7)	628 (38.3)	1.65 (1.37-1.99)
Injection equipment sharing past 30 d	375 (61.2)	739 (45.1)	1.92 (1.59-2.32)
Drug treatment ever	516 (80.9)	1327 (78.2)	1.18 (0.94-1.48)
Traded sex for money past 30 d	65 (12.4)	125 (9.5)	1.34 (0.98-1.85)
Sex without a condom past 30 d	342 (65.6)	763 (59.1)	1.32 (1.07-1.63)
Received buprenorphine from “doctor or program” past 30 d	123 (19.4)	153 (9.1)	2.42 (1.87-3.13)
Could not get buprenorphine from “doctor or program” past 6 mo	159 (25.0)	274 (16.8)	1.65 (1.32-2.06)
Current drug preference^b^			
Heroin	268 (41.7)	658 (38.1)	1 [Reference]
Street fentanyl or carfentanil	16 (2.5)	36 (2.1)	1.09 (0.60-2.00)
Opiate painkiller	54 (8.4)	105 (6.1)	1.26 (0.88-1.81)
Synthetic opioid	14 (2.2)	5 (0.3)	6.88 (2.45-19.27)
Buprenorphine	45 (7.0)	16 (0.9)	6.91 (3.84-12.43)
Methadone	8 (1.3)	26 (1.5)	0.76 (0.34-1.69)
Prescription anxiety drug	11 (1.7)	20 (1.2)	1.35 (0.64-2.86)
Cocaine or crack	32 (5.0)	99 (5.7)	0.79 (0.52-1.21)
Methamphetamine, crystal methamphetamine, or amphetamine	183 (28.5)	714 (41.3)	0.63 (0.51-0.78)
Other	10 (1.6)	41 (2.4)	0.60 (0.30-1.21)
Heavy alcohol consumption (≥4/5 drinks in 1 d) in past 30 d^c^	369 (57.8)	764 (44.9)	1.69 (1.40-2.03)
Cigarette smoking past 30 d	594 (92.7)	1569 (91.1)	1.26 (0.90-1.76)
Ever experienced an overdose	379 (60.5)	830 (49.1)	1.58 (1.31-1.91)

Abbreviation: OR, odds ratio.

^a^ Participants were recruited across 10 US states, including Illinois, Kentucky, New England (New Hampshire, Vermont, and Massachusetts), North Carolina, Oregon, Ohio, West Virginia, and Wisconsin.

^b^ Opiate painkillers include oxycodone, Percocet, Percodan, OxyContin, hydrocodone, Vicodin, Lorcet, Lortab, Norco, morphine, Dilaudid, Opana, T3s, and fentanyl patch. Synthetic opioids include U-47700, U4, or pink. Prescription anxiety drugs include Xanax, Valium, and Klonopin. Other drugs include gabapentin, Neurontin, clonidine, or any other drug.

^c^ The cutoff of more than 4 to 5 drinks per month for heavy alcohol consumption varied according to respondent reported sex at birth (female: >4 drinks/mo; male: >5 drinks/mo).

## Discussion

In this cross-sectional study, people who injected buprenorphine intended for sublingual use more commonly reported sex- and drug-related behaviors associated with sexually transmitted and bloodborne infection transmission and overdose risk. Further research is needed to confirm these associations in other settings. Study limitations include not being able to establish causality and that variables were asked across different time frames. Prior studies have begun exploring reasons for injecting buprenorphine.^2,6^ Future research should explore buprenorphine injection trajectories, reasons behind the behavior, and ways to provide alternatives to injecting buprenorphine while maintaining medications for OUD treatment engagement. These findings suggest the need for open conversations between patients and clinicians providing OUD treatment about formulations of injectable buprenorphine that can be prescribed, as well as pre-exposure prophylaxis and HIV and hepatitis C testing and treatment to reduce potential risks for infectious disease transmission and overdose.

## Notes

Supplement.
Data Sharing Statement
