# Supplementary material for: Buprenorphine Injection Among Rural Persons Who Inject Drugs
Source: JAMA Netw Open. 2024 Dec 11;7(12):e2450108. doi: 10.1001/jamanetworkopen.2024.50108 (PMC11635541; doi:10.1001/jamanetworkopen.2024.50108)
Supplement: Supplement. — Data Sharing Statement [file jamanetwopen-e2450108-s001.pdf]

Data Sharing Statement

Zinsli. Buprenorphine Injection Among Rural Persons Who Inject Drugs. *JAMA Netw Open*. Published online December 11, 2024. doi:10.1001/jamanetworkopen.2024.50108

Data
Data available: No
